# Synthesis of Novel 7-Substituted-5-phenyl-[1,2,4]triazolo[1,5-a] Pyrimidines with Anticonvulsant Activity

**Published:** 2012

**Authors:** Nan Jiang, Xian-Qing Deng, Fu-Nan Li, Zhe-Shan Quan

**Affiliations:** a *College of Pharmacy, Yanbian University, No. 977, Park road, Yanji, Jilin, 133002, China.*; b *Department of Pharmacy, Medical College of Xiamen University, No. 168, Daxue Rd, Xiamen, Fujian, 361005, China.*

**Keywords:** Synthesis, Triazole, Pyrimidine, Anticonvulsant, Maximal electroshock

## Abstract

Considerable interest has been focused on the triazole structure, which has been known to possess a broad spectrum of biological activities such as antitumor, anti-inflammatory, antimicrobial, antiviral, and anticonvulsant activities. Before this, several heterocyclic compounds containing triazole were synthesized that had shown considerable anticonvulsant activity. As part of our continuous research in this area, we have synthesized several new 7-substituted-5-phenyl-[1,2,4] triazolo[1,5-*a*] pyrimidines (compounds 3a-3i, 5a-5j) through incorporating triazole moiety into the pyrimidine ring, which are expected to have the synergistic effect in dealing with the epilepsy. Their anticonvulsant activities were measured through the Maximal electroshock (MES) test. Carbamazepine and valproate were considered as positive control drugs with anticonvulsant effects [ED_50_ = 11.8 and 272 mg/Kg]. Amongst the compounds tested, compound 3f, 7-(heptyloxy)-5-phenyl-[1,2,4] triazolo[1,5-*a*] pyrimidine, showed potent anticonvulsant activity with ED_50_ 84.9 mg/Kg, which was weaker than carbamazepine, but better than valproate.

## Introduction

Epilepsy, one of the most frequent neurological afflictions in men characterized via excessive temporary neuronal discharges resulting in uncontrolled convulsion, inflicts more than 60 million people worldwide (1, 2). Despite the development of several new anticonvulsants, the treatment of epilepsy remains still inadequate. It is roughly estimated that up to 28-30% of patients are poorly treated with the available antiepileptic drugs (AEDs) (3, 4). Moreover, many AEDs have serious side effects (5-10) and lifelong medication may be required. Therefore, there is a continuing demand for new anticonvulsant agents with more selectivity and lower toxicity.

In the effort to get those agents, we have reported (11-17) several heterocyclic compounds containing triazole, which have shown considerable anticonvulsant activities. From the currently used AEDs, the major characteristics important in newly synthesized compounds are the inclusion of a hydrophobic site and H-bond donors/acceptors. With respect to the compounds we reported previously, the hydrophobic site is obviously the phenyl group and the substituents on it, and the H-bond acceptor is the triazole.

As a part of our continuous research in this area, we have designed and synthesized several new 7-substituted-5-phenyl-[1,2,4] triazolo[1,5-*a*] pyrimidines (compound 3a-3i, 5a-5j) through incorporating triazole moiety into the pyrimidine ring, which are expected to have the synergistic effect in dealing with the epilepsy. In this series of compounds, the 5^th^ position phenyl group and the 7^th^ position substitutes is the hydrophobic site, which could contribute to the traverse blood-brain barrier and also the H-bond acceptor is still the triazole.

**Figure 1:**
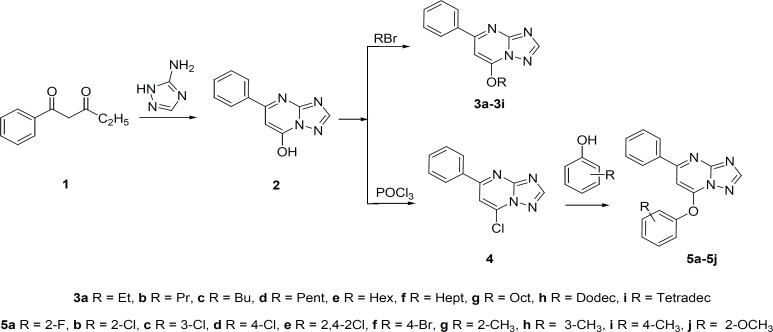
The synthesis route of target compounds

New compounds were synthesized according to Figure 1. 

The reaction of 1-phenylpentane-1,3-dione (1) with 2*H*-1,2,4-triazol-3-amine under 160oC afforded 5-phenyl-[1,2,4] triazolo[1,5-a]pyrimidin-7-ol (2). The alkylation of compound 2 with appropriate alkyl bromide in Dimethylformamide (DMF) in the presence of NaOH and KI at 80oC afforded 7-alkoxy-5-phenyl-[1,2,4]triazolo[1,5-a]pyrimidine derivatives (compound 3a-3i). Compound 4 was obtained by means of boiling compound 3 with excessive POCl_3_. The refluxing of compound 4 with appropriate substituted phenol in acetonitrile in the presence of sodium hydroxide gave substituted 7-phenoxy-5-phenyl-[1,2,4] triazolo[1,5-a] pyrimidine derivatives (compound 5a-5j) (Figure 1).

The anticonvulsant activity of synthesized compounds was determined through the maximal electroshock seizure (MES), which is one of the animal seizure models most widely used in the search for new AEDs (18). 

## Experimental

Preparation of compounds

*5-Phenyl-[1,2,4]triazolo[1,5-a]pyrimidin-7-ol (compound 2)*

The 1-phenylpentane-1,3-dione (compound 1) (3.00 g, 15.6 mmol) and 2*H*-1,2,4-triazol-3-amine (2.00 g, 23.8 mmol) were reacted at 160°C for 2 h with no solvent. After cooling, the mixture was filtered and washed with dichloromethane to afford compound 2 in 92% yield. M.p. 201-203°C, IR (KBr) cm^-1^: 1616 (C=N), 1537 (C=C), 1194 (N–N). MS m/z 213 (M+1). ^1^H-NMR (DMSO-*d*_6_, 300 MHz) δ 6.35 (s, 1H, H-6), 7.48 (s, 1H, -OH), 7.53-7.55 (m, 3H, Ph-H), 7.89-7.93 (m, 2H, Ph-H), 8.38 (s, 1H, H-2). Anal.Calcd. for C_11_H_8_N_4_O: C, 62.26; H, 3.80; N, 26.40. Found: C, 62.12; H, 3.65; N, 26.56.

*7-Chloro-5-phenyl-[1,2,4]triazolo[1,5-a]pyrimidine (compound 4)*

Compound 2 (1.00 g, 4.72 mmol) were placed into a 100 mL round-bottomed flask containing 30 mL of POCl_3_ equipped with a reflux condenser connected with a drying tube. The mixture was stirred and heated at 100°C for 3 h. Then, most of the solvent was removed under reduced pressure and the mixture was poured into ice-water. The precipitate was filtered and washed with water and recrystallized from CH_3_CO_2_C_2_H_5_ to afford compound 4 in 85% yield. M.p. 146-148°C, IR (KBr) cm^-1^: 1636 (C=N), 1557 (C=C), 1213 (N–N). MS m/z 231 (M+1). ^1^H-NMR (CDCl_3_, 300 MHz) δ7.72 (s, 1H, H-6), 7.56-7.66 (m, 3H, Ph-H), 8.19-8.22 (m, 2H, Ph-H), 8.59 (s, 1H, H-2). Anal.Calcd. for C_11_H_7_ClN_4_: C, 57.28; H, 3.06; N, 24.29. Found: C, 57.13; H, 3.22; N, 24.44.

*7-Alkoxy-5-phenyl-[1,2,4]triazolo[1,5-a]pyrimidine derivatives (compounds 3a-3i)*

Compound 2 (0.30 g, 1.42 mmol) and NaOH (0.06 g, 1.50 mmol) were placed into a 100 mL round-bottomed flask containing 30 mL of DMF. After the mixture was stirred and heated at 80°C for 3 h, various kinds of substituted alkyl bromide (1.68 mmol) and KI (1.68 mmol) were added. After stirring for about 16 h, the solvent was removed under reduced pressure. The mixture was extracted twice with dichloromethane. The dichloromethane layer was dried over anhydrous MgSO_4_. The evaporation of the solvents got a crude product, which was purified through silica gel column chromatography with CH_2_Cl_2_ and CH_3_OH (30:1) to obtain compounds 3a-3i. The yield, melting point and spectral data of each compound were given below.

*Substituted 7-phenoxy -5-phenyl-[1,2,4]triazolo[1,5-a]pyrimidine derivatives (compounds5a-5j)*

Various kinds of Substituted phenol (1.50 mmol) and NaOH (0.06 g, 1.50 mmol) were placed into a 100 mL round-bottomed flask containing 30 mL of CH_3_CN. After the mixture was stirred and heated at 80°C for 1 h, Compound 4 (0.3 g, 1.30 mmol) was added. Following the stirring for about 5 h, the solvent was removed under reduced pressure. The mixture was extracted twice with dichloromethane. The dichloromethane layer was dried over anhydrous MgSO_4_. The evaporation of solvents get a crude product, which was purified by silica gel column chromatography with CH_2_Cl_2_ and CH_3_OH (25:1) to obtain compounds 5a-5j. The yield, melting point and spectral data of each compound were given below.

*7-Ethoxy-5-phenyl-[1,2,4]triazolo[1,5-a]pyrimidine (compound 3a)*

M.p. > 270°C; yield 27.5%; ^1^H-NMR (CDCl_3_, 300 MHz) δ 1.63 (t, 3H, *J *= 7.3 Hz, -CH_3_), 4.28 (q, 2H, *J *= 7.3 Hz, -OCH_2_-), 6.72 (s, 1H, H-6), 7.47-7.49 (m, 3H, Ph-H), 8.00-8.03 (m, 2H, Ph-H), 8.15 (s, 1H, H-2); IR (KBr) cm^-1^: 1680 (C=N), 1537 (C=C), 1146 (N–N); MS m/z 241 (M+1); Anal.Calcd. for C_13_H_12_N_4_O: C, 64.99; H, 5.03; N, 23.32. Found: C, 64.86; H, 5.12; N, 23.50.

*7-Propoxy-5-phenyl- [1,2,4]triazolo[1,5-a]pyrimidine (compound 3b)*

M.p. 228-230°C; yield 39.7%. ^1^H-NMR (CDCl_3_, 300 MHz) δ 1.07 (t, 3H, *J *= 7.4 Hz, -CH_3_), 1.96-2.08 (m, 2H, -CH_2_-), 4.18 (t, 2H, *J *= 7.2 Hz, -OCH_2_-), 6.72 (s, 1H, H-6), 7.47-7.49 (m, 3H, Ph-H), 8.00-8.03 (m, 2H, Ph-H), 8.11 (s, 1H, H-2); IR (KBr) cm^-1^: 1681 (C=N), 1538 (C=C), 1146 (N–N); MS m/z 255 (M+1); Anal.Calcd. for C_14_H_14_N_4_O: C, 66.13; H, 5.55; N, 22.03. Found: C, 66.32; H, 5.66; N, 22.11.

*7-Butoxy-5-phenyl-[1,2,4]triazolo[1,5-a]pyrimidine (compound 3c)*

M.p. 209-210°C; yield 42.2%. ^1^H-NMR (CDCl_3_, 300 MHz) δ 1.03 (t, 3H, *J *= 7.3 Hz, -CH_3_), 1.40-1.52 (m, 2H, -CH_2_-), 1.90-2.01 (m, 2H, -CH_2_-), 4.21 (t, 2H, *J *= 7.2 Hz, -OCH_2_-), 6.72 (s, 1H, H-6), 7.47-7.49 (m, 3H, Ph-H), 8.00-8.03 (m, 2H, Ph-H), 8.13 (s, 1H, H-2); IR (KBr) cm^-1^: 1681 (C=N), 1540 (C=C), 1150 (N–N); MS m/z 269 (M+1); Anal.Calcd. for C_15_H_16_N_4_O: C, 67.15; H, 6.01; N, 20.88. Found: C, 67.28; H, 6.13; N, 20.99.

*7-Pentyloxy-5-phenyl-[1,2,4]triazolo[1,5-a]pyrimidine (compound 3d)*

M.p. 168-171°C; yield 39.6%. ^1^H-NMR (CDCl_3_, 300 MHz) δ 0.94 (t, 3H, *J *= 6.5 Hz, -CH_3_), 1.41-1.44 (m, 4H, -(CH_2_)_2_-), 1.95-2.00 (m, 2H, -CH_2_-), 4.20 (t, 2H, *J *= 7.3 Hz, -OCH_2_-), 6.73 (s, 1H, H-6), 7.47-7.49 (m, 3H, Ph-H), 8.00-8.03 (m, 2H, Ph-H), 8.11 (s, 1H, H-2); IR (KBr) cm-1: 1683 (C=N), 1543 (C=C), 1153 (N–N); MS m/z 283 (M+1); Anal.Calcd. for C_16_H_18_N_4_O: C, 68.06; H, 6.43; N, 19.84. Found: C, 68.23; H, 6.61; N, 19.62.

*7-Hexyloxy-5-phenyl-[1,2,4]triazolo[1,5-a]pyrimidine (compound 3e)*

M.p. 157-160°C; yield 29.1%. ^1^H-NMR (CDCl_3_, 300 MHz) δ 0.90 (t, 3H, *J *= 6.9 Hz, -CH_3_), 1.35-1.39 (m, 6H, -(CH_2_)_3_-), 1.95-1.99 (m, 2H, -CH_2_-), 4.20 (t, 2H, *J *= 7.3 Hz, -OCH_2_-), 6.72 (s, 1H, H-6), 7.47-7.49 (m, 3H, Ph-H), 8.00-8.03 (m, 2H, Ph-H), 8.12 (s, 1H, H-2); IR (KBr) cm^-1^: 1683 (C=N), 1543 (C=C), 1152 (N–N); MS m/z 297 (M+1); Anal.Calcd. for C_17_H_20_N_4_O: C, 68.89; H, 6.80; N, 18.90. Found: C, 68.71; H, 6.93; N, 18.75.

*7-Heptyloxy-5-phenyl-[1,2,4]triazolo[1,5-a]pyrimidine (compound 3f)*

M.p. 139-141°C; yield 33.7%.^ 1^H-NMR (CDCl_3_, 300 MHz) δ 0.89 (t, 3H, *J *= 6.9 Hz, -CH_3_), 1.30-1.41 (m, 8H, -CH_2_-CH_2_-CH_2_-CH_2_-), 1.95-1.99 (m, 2H, -CH_2_-), 4.20 (t, 2H, *J *= 7.2 Hz, -OCH_2_-), 6.73 (s, 1H, H-6), 7.48-7.50 (m, 3H, Ph-H), 8.00-8.02 (m, 2H, Ph-H), 8.13 (s, 1H, H-2); IR (KBr) cm^-1^: 1684 (C=N), 1546 (C=C), 1158 (N–N); MS m/z 311 (M+1); Anal.Calcd. for C_18_H_22_N_4_O: C, 69.65; H, 7.14; N, 18.05. Found: C, 69.42; H, 7.02; N, 18.21.

*7-Octyloxy-5-phenyl-[1,2,4]triazolo[1,5-a]pyrimidine (compound 3g)*

M.p. 125-128oC; yield 32.8%. _1_H-NMR (CDCl_3_, 300 MHz) δ 0.87 (t, 3H, *J *= 6.9 Hz, -CH_3_), 1.27-1.39 (m, 10H, -(CH_2_)_5_-), 1.94-1.97 (m, 2H, -CH_2_-), 4.20 (t, 2H, *J *= 7.2 Hz, -OCH_2_-), 6.73 (s, 1H, H-6), 7.47-7.49 (m, 3H, Ph-H), 8.00-8.02 (m, 2H, Ph-H), 8.11 (s, 1H, H-2); IR (KBr) cm_-1_: 1684 (C=N), 1545 (C=C), 1156 (N–N); MS m/z 325 (M+1); Anal.Calcd. for C_19_H_24_N_4_O: C, 70.34; H, 7.46; N, 17.27. Found: C, 70.52; H, 7.33; N, 17.45.

*7-Dodecyloxy-5-phenyl-[1,2,4]triazolo[1,5-a]pyrimidine (compound 3h)*

M.p. 117-119oC; yield 32.1%. ^1^H-NMR (CDCl_3_, 300 MHz) δ 0.88 (t, 3H, *J *= 6.6 Hz, -CH_3_), 1.25-1.40 (m, 18H, -(CH_2_)_9_-), 1.95-1.99 (m, 2H, -CH_2_-), 4.19 (t, 2H, *J *= 7.2 Hz, -OCH_2_-), 6.73 (s, 1H, H-6), 7.47-7.49 (m, 3H, Ph-H), 8.01-8.02 (m, 2H, Ph-H), 8.09 (s, 1H, H-2); IR (KBr) cm^-1^: 1688 (C=N), 1551 (C=C), 1160 (N–N); MS m/z 381 (M+1); Anal.Calcd. for C_23_H_32_N_4_O: C, 72.60; H, 8.48; N, 14.72. Found: C, 72.82; H, 8.66; N, 14.50.

*7-Tetradecyloxy-5-phenyl-[1,2,4]triazolo[1,5-a]pyrimidine (compound 3i)*

M.p. 106-109°C; yield 41.6%. ^1^H-NMR (CDCl_3_, 300 MHz) δ 0.88 (t, 3H, *J *= 6.6 Hz, -CH_3_), 1.25-1.40 (m, 22H, -(CH_2_)_11_-), 1.94-1.97 (m, 2H, -CH_2_-), 4.20 (t, 2H, *J *= 7.2 Hz, -OCH_2_-), 6.72 (s, 1H, H-6), 7.47-7.49 (m, 3H, Ph-H), 8.00-8.03 (m, 2H, Ph-H), 8.11 (s, 1H, H-2); IR (KBr) cm^-1^: 1689 (C=N), 1551 (C=C), 1159 (N–N); MS m/z 409 (M+1); Anal.Calcd. for C_25_H_36_N_4_O: C, 73.49; H, 8.88; N, 13.71. Found: C, 73.71; H, 8.79; N, 13.93.

*7-(4-Fluorophenoxy)-5-phenyl-[1,2,4]triazolo[1,5-a]pyrimidine (compound 5a)*

M.p. 160-162°C; yield 61.1%. ^1^H-NMR (CDCl_3_, 300 MHz) δ 6.59 (s, 1H, H-6), 7.25-7.30 (m, 2H, Ph-H), 7.36 (d, 2H, *J *= 7.9 Hz, Ph-H), 7.46-7.54 (m, 3H, Ph-H), 8.01 (d, 2H, *J *= 7.9 Hz, Ph-H), 8.55 (s, 1H, H-2); IR (KBr) cm^-1^: 1628 (C=N), 1543 (C=C), 1196 (N–N); MS m/z 307 (M+1); Anal.Calcd. for C_17_H_11_FN_4_O: C, 66.66; H, 3.62; N, 18.29. Found: C, 66.82; H,3.46; N, 18.34.

*7-(2-Chlorophenoxy)-5-phenyl-[1,2,4]triazolo[1,5-a]pyrimidine (compound 5b)*

M.p. 158-161°C; yield 63.5%. ^1^H-NMR (CDCl_3_, 300 MHz) δ 6.49 (s, 1H, H-6), 7.42-7.49 (m, 6H, Ph-H), 7.63-7.66 (m, 1H, Ph-H), 7.99-8.02 (m, 2H, Ph-H), 8.57 (s, 1H, H-2); IR (KBr) cm^-1^: 1627 (C=N), 1542 (C=C), 1208 (N–N); MS m/z 323 (M+1); Anal.Calcd. for C_17_H_11_ClN_4_O: C, 63.26; H, 3.44; N, 17.36. Found: C, 63.02; H, 3.29; N, 17.45.

*7-(3-Chlorophenoxy)-5-phenyl-[1,2,4]triazolo[1,5-a]pyrimidine (compound 5c)*

M.p. 144-147°C; yield 28.6%. ^1^H-NMR (CDCl_3_, 300 MHz) δ 6.70 (s, 1H, H-6), 7.71-7.54 (m, 7H, Ph-H), 8.03-8.05 (m, 2H, Ph-H), 8.60 (s, 1H, H-2); IR (KBr) cm^-1^: 1625 (C=N), 1544 (C=C), 1207 (N–N); MS m/z 323 (M+1); Anal.Calcd. for C_17_H_11_ClN_4_O: C, 63.26; H, 3.44; N, 17.36. Found: C, 63.12; H, 3.31; N, 17.52.

*7-(4-Chlorophenoxy)-5-phenyl-[1,2,4]triazolo[1,5-a]pyrimidine (compound 5d)*

M.p. 146-148°C; yield 62.5%. ^1^H-NMR (CDCl_3_, 300 MHz) δ 6.62 (s, 1H, H-6), 7.33 (d, 2H, *J *= 8.0 Hz, Ph-H), 7.46-7.57 (m, 5H, Ph-H), 8.02 (d, 2H, *J *= 8.0 Hz, Ph-H), 8.54 (s, 1H, H-2); IR (KBr) cm^-1^: 1625 (C=N), 1547 (C=C), 1210 (N–N); MS m/z 323 (M+1); Anal.Calcd. for C_17_H_11_ClN_4_O: C, 63.26; H, 3.44; N, 17.36. Found: C, 63.19; H, 3.38; N, 17.49. 

*7-(2,4-Dichlorophenoxy)-5-phenyl-[1,2,4]triazolo[1,5-a]pyrimidine (compound 5e)*

M.p. 211-214°C; yield 41.9%. ^1^H-NMR (CDCl_3_, 300 MHz) δ 6.50 (s, 1H, H-6), 7.32-7.51 (m, 5H, Ph-H), 7.65-7.66 (m, 1H, Ph-H), 8.01-8.03 (m, 2H, Ph-H), 8.57 (s, 1H, H-2); IR (KBr) cm^-1^: 1626 (C=N), 1545 (C=C), 1204 (N–N); MS m/z 357 (M+1); Anal.Calcd. for C_17_H_10_Cl_2_N_4_O: C, 57.16; H, 2.82; N, 15.69. Found: C, 57.32; H, 2.70; N, 15.84.

*7-(4-Bromophenoxy)-5-phenyl-[1,2,4]triazolo[1,5-a]pyrimidine (compound 5f)*

M.p. 143-145°C; yield 55.0%. ^1^H-NMR (CDCl_3_, 300 MHz) δ 6.63 (s, 1H, H-6), 7.27 (d, 2H, *J *= 7.4 Hz, Ph-H), 7.47-7.53 (m, 3H, Ph-H), 7.72 (d, 2H, *J *= 7.4 Hz, Ph-H), 8.01-8.04 (m, 2H, Ph-H), 8.55 (s, 1H, H-2); IR (KBr) cm^-1^: 1622 (C=N), 1539 (C=C), 1211 (N–N); MS m/z 367 (M+1); Anal.Calcd. for C_17_H_11_BrN_4_O: C, 55.61; H, 3.02; N, 15.26. Found: C, 55.75; H, 3.13; N, 15.39.

*7-(2-Methylphenoxy)-5-phenyl-[1,2,4]triazolo[1,5-a]pyrimidine (compound 5g)*

M.p. 118-120°C; yield 61.5%. ^1^H-NMR (CDCl_3_, 300 MHz) δ 2.29 (s, 3H, -CH_3_), 6.49 (s, 1H, H-6), 7.28-7.29 (m, 1H, Ph-H), 7.36-7.48 (m, 6H, Ph-H), 7.96-8.00 (m, 2H, Ph-H), 8.55 (s, 1H, H-2); IR (KBr) cm^-1^: 1612 (C=N), 1544(C=C), 1165 (N–N); MS m/z 303 (M+1); Anal.Calcd. for C_18_H_14_N_4_O: C, 71.51; H, 4.67; N, 18.53. Found: C, 71.66; H, 4.59; N, 18.69.

*7-(3-Methylphenoxy)-5-phenyl-[1,2,4]triazolo[1,5-a]pyrimidine (compound 5h)*

M.p. 123-126°C; yield 61.6%. ^1^H-NMR (CDCl_3_, 300 MHz) δ 2.46 (s, 1H, CH_3_), 6.64 (s, 1H, H-6), 7.15-7.49 (m, 7H, Ph-H), 8.00-8.03 (m, 2H, Ph-H), 8.56 (s, 1H, H-2); IR (KBr) cm^-1^: 1611 (C=N), 1545 (C=C), 1163 (N–N);MS m/z 303 (M+1); Anal.Calcd. for C_18_H_14_N_4_O: C, 71.51; H, 4.67; N, 18.53. Found: C, 71.65; H, 4.79; N, 18.66.

*7-(4-Methylphenoxy)-5-phenyl- [1,2,4]triazolo[1,5-a]pyrimidine (compound 5i)*

M.p. 167-169°C; yield 50.9%. ^1^H-NMR (CDCl_3_, 300 MHz) δ 2.46 (s, 3H, -CH_3_), 6.61 (s, 1H, H-6), 7.25 (d, 2H, *J *= 8.0 Hz, Ph-H), 7.34-7.49 (m, 5H, , Ph-H), 7.99-8.02 (m, 3H, Ph-H), 8.01 (d, 2H, *J *= 8.0 Hz, Ph-H), 8.53 (s, 1H, H-2); IR (KBr) cm^-1^: 1614 (C=N), 1547 (C=C), 1165 (N–N); MS m/z 303 (M+1); Anal.Calcd. for C_18_H_14_N_4_O: C, 71.51; H, 4.67; N, 18.53. Found: C, 71.36; H, 4.82; N, 18.66.

*7-(2-Methoxyphenoxy)-5-phenyl-[1,2,4]triazolo[1,5-a]pyrimidine (compound 5j)*

M.p. 166-168°C; yield 43.5%. ^1^H-NMR (CDCl_3_, 300 MHz) δ 3.80 (s, 3H, -OCH_3_), 6.52 (s, 1H, H-6), 7.12-7.15 (m, 2H, Ph-H), 7.36-7.48 (m, 5H, Ph-H), 7.99-8.02 (m, 2H, Ph-H), 8.55 (s, 1H, H-2); IR (KBr) cm^-1^: 1610 (C=N), 1542(C=C), 1163(N–N); MS m/z 307 (M+1); Anal.Calcd. for C_18_H_14_N_4_O_2_: C, 67.91; H, 4.43; N, 17.60. Found: C, 67.76; H, 4.37; N, 17.74.

*Pharmacology*

Kunming mice (supplied from the Laboratory of Animal Research, Yanbian University, China) weighting 18-22 g were used for pharmacological study. Animals were allowed free access to food and water except during the experiment and housed at controlled room temperature with 12 h light/dark schedule. All compounds were dissolved in Dimethyl sulfoxide DMSO with the injection volume of 0.05 mL per 20 g, which had no effect on the test system.

*Anticonvulsant activity in the maximal electroshock seizure (MES) test*

Anticonvulsant activity of the synthesized compounds was determined through the evaluation of the compounds ability to protect mice against MES-induced seizures. The MES test was carried out by the methods described in the ADD of the National Institutes of Health (USA) (18, 19). Seizures were elicited with a 60 Hz alternating current of 50 mA intensity in mice. The current was applied via corneal electrodes for 0.2 s. Protection against the spread of MES-induced seizures was defined as the abolition of tonic maximal extension of the hind leg. At 30 min after the administration of compounds, the activities were evaluated in MES test. In phase-I screening, each compound was administered at the dose levels of 100 mg/Kg for evaluating the preliminary anticonvulsant activity. For the determination of median effective dose (ED_50_) and the median toxic dose (TD_50_), the phase-II screening was prepared. Groups of 10 mice were given a range of intraperitoneal doses of the tested compound until at least three points were established in the range of 10-90% seizure protection or minimal observed neurotoxicity. From the plot of this data, the respective ED_50_ and TD_50_ values, 95% confidence intervals, slope of the regression line, and the standard error of the slope were calculated with the statistical software SPSS 13.0.

*Neurotoxicity screening (NT)*

The neurotoxicity of the compounds was measured in mice through the rotarod test (19, 20). The mice were trained to stay on a rotarod with a diameter of 3.2 cm that rotates at 10 rpm. Trained animals were given IP-injection of the test compounds. Neurotoxicity was indicated by the inability of the animal to maintain equilibrium on the rod for at least 1 min in each of the trials.

## Results and Discussion

The maximal electroshock (MES) model was carried out to preliminary evaluate (phase I) the prepared compounds (compounds 3a-3i, 5a-5j) for the anticonvulsant activity. As shown in Table 1, some of the compounds were active in the MES test in dose of 100 mg/Kg, the indicative of their ability to prevent seizure spread. Among alkoxy group substituted derivatives (compounds 3a-3i), compounds 3c-3h showed protection against MES-induced seizure in varying degrees at the dose of 100 mg/Kg. Compound 3f was the best one as its complete protection. 


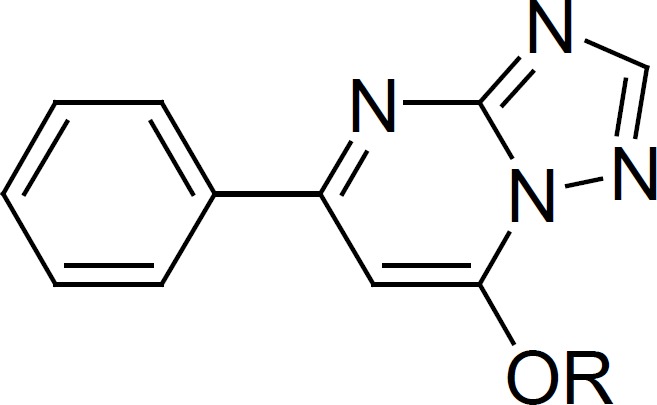


**Table 1:** The phase I data of compounds 3a-3i, 5a-5j in the MES in mice (IP).

Compds.	R	MES^a^ (100 mg/Kg)
**3a**	-C2H5	0/6
**3b**	-C3H7	0/6
**3c**	-C4H9	1/6
**3d**	-C5H11	2/6
**3e**	-C6H13	2/6
**3f**	-C7H15	6/6
**3g**	-C8H17	4/6
**3h**	-C12H25	3/6
**3i**	-C14H29	0/6
**5a**	-C6H4(*p*-F)	0/6
**5b**	-C6H4(*o*-Cl)	0/6
**5c**	-C6H4(*m*-Cl)	0/6
**5d**	-C6H4(*p*-Cl)	0/6
**5e**	-C6H3(*2,4-*Cl2)	0/6
**5f**	-C6H4(*p*-Br)	0/6
**5g**	-C6H4(*o*-CH3)	0/6
**5h**	-C6H4(*m*-CH3)	0/6
**5i**	-C6H4(*p*-CH3)	0/6
**5j**	-C6H4(*o*-OCH3)	0/6

^a^Maximal electroshock test (number of animals protected/number of animals tested) (the number of mice is six).

Among phenoxy group substituted derivatives (compounds 5a-5j), none showed protection against MES-induced seizure at dose of 100 mg/Kg. The weak activity of 5a-5j may be due to the big size of their phenoxy group in 7^th^ position, which may reduce the affinity between the triazole and receptor. For the alkoxy substituted derivatives (compounds 3a-3i), the length of the alkoxyl chain appeared to have impact on the anticonvulsant activity of them. From 3c to 3f, as the alkoxyl chain length increased, the anticonvulsant activity was gradually increased with the compound 3f (with the *n*-heptyloxy group in 7^th^ position) being the most active compound. The trend reversed, however, when the alkyl chain had more than seven carbon atoms (compounds 3f-3h). Obviously, the activity curve of the alkyl chain substituted derivatives is bell-shaped with a maximum activity peak. Compound 3f, with the maximum activity in this series of compounds, reflected the optimal partition coefficient associated with the easiest crossing of the biological membranes and the optimal stereo configuration.

As a result of preliminary screening, compound 3f was subjected to phase II trials for the quantification of its anticonvulsant activity (indicated with ED_50_) and neurotoxicity (indicated with TD_50_) in mice. Results of the quantitative test for 3f, along with the data on the standard drugs valproate and carbamazepine, are reported in Table 2. 

**Table 2:** Phase II quantitative anticonvulsant data in mice (IP).

**Compds.**	**R**	**ED**_50_ **(mg·Kg** ^-1^ **)**	**TD**_50_ **(mg·Kg** ^-1^ **)**	**PI(TD**_50_ **/ED**_50_ **)**
3f	-C_7_H_15_	84.9 (74.3-97.0)	509.2 (476.3-544.4)	6.0
Valproate	-	272.0 (247.1-338.8)	426.1 (369.4-450.3)	1.6
Carbamazepine	-	11.8 (8.5-16.4)	76.1 (55.8-103.7)	6.4

Compound 3f, which gave an ED_50_ value of 84.9 mg/Kg, displayed a weaker anticonvulsant activity compared to carbamazepine (ED_50_ = 11.8 mg/Kg), but a higher activity compared to valproate (ED_50_ = 272 mg/Kg). Moreover, 3f showed a higher TD_50_-value (TD_50_ = 509.2) in comparison to carbamazepine (TD_50_ = 76.1) and valproate (TD_50 _= 426), which make its PI value close to carbamazepine and higher than valproate.

For further exploring the anticonvulsant activity of these compounds, PTZ-induced seizure model was made to 3f. As shown in Table 3, no protection was observed at the 100 mg/Kg and 200 mg/Kg doses, which suggested that compound 3f cannot be against the seizure induced by PTZ. PTZ has been reported to produce seizures by inhibiting γ-aminobutyric acid (GABA) neurotransmission. GABA is the main inhibitory neurotransmitter in the brain, and is widely implicated in epilepsy. From the data of Table 3, it is speculated that the mechanism of the novel compounds’ action may not involve in the GABAergic neurotransmission.

**Table 3:** PTZ-induced seizure test data of 3f in mice (IP)

**Compds.**	**Dose (mg/mg)**	**Number of animals**	**Number of seizures**
Control	-	10	10
3f	100	10	10
3f	200	10	10

In conclusion, most of these compounds possessed the weak anticonvulsant effect under dose of 100 mg/Kg, which did not achieve the previously designed expectation, and showed lower activity compared to the compounds with similar chemical structures previously synthesized in our laboratory.
